# Appressoria—Small but Incredibly Powerful Structures in Plant–Pathogen Interactions

**DOI:** 10.3390/ijms24032141

**Published:** 2023-01-21

**Authors:** Ting-Ting Shi, Guo-Hong Li, Pei-Ji Zhao

**Affiliations:** State Key Laboratory for Conservation and Utilization of Bio-Resources in Yunnan, School of Life Sciences, Yunnan University, Kunming 650091, China

**Keywords:** appressoria, interaction, plant, pathogenic fungi

## Abstract

Plant-pathogenic fungi are responsible for many of the most severe crop diseases in the world and remain very challenging to control. Improving current protection strategies or designating new measures based on an overall understanding of molecular host–pathogen interaction mechanisms could be helpful for disease management. The attachment and penetration of the plant surface are the most important events among diverse plant–fungi interactions. Fungi evolved as small but incredibly powerful infection structure appressoria to facilitate attachment and penetration. Appressoria are indispensable for many diseases, such as rusts, powdery mildews, and blast diseases, as well as devastating oomycete diseases. Investigation into the formation of plant–pathogen appressoria contributes to improving the understanding of the molecular mechanisms of plant–pathogen interactions. Fungal host attachment is a vital step of fungal pathogenesis. Here, we review recent advances in the molecular mechanisms regulating the formation of appressoria. Additionally, some biocontrol agents were revealed to act on appressorium. The regulation of fungal adhesion during the infective process by acting on appressoria formation is expected to prevent the occurrence of crop disease caused by some pathogenic fungi.

## 1. Introduction

Fungal spores take full advantage of natural conditions, such as insect vectors, wind, or water, to spread and reach their final destination host. Settling on the plant surface is the first task for successful infection. These spores are capable of sticking tightly to host or non-host plant surfaces. Firm attachment protects the spores from being removed from the surface before penetration [1]. Spores secrete an adhesive extracellular matrix that allows them to attach firmly to the host surface. Hamer et al. have shown that the *Magnaporthe oryzae* spore wall ruptures upon the hydration of the conidia and releases spore-tip mucilage stored in a periplasmic compartment at the conidial apex to accomplish attachment to various surfaces [2]. Subsequently, the spore tapering end emerges as a single, polarized hypha, which grows along the host plant surface, and finally differentiates into the dome-shaped appressorium to force its way through the cell wall by localized physical pressure [3,4]. Hyphae show polarized cell growth and grow along the host plant surface, which depends on the recognition of distinct physical (surface hardness, hydrophobicity) and chemical (cutin monomers, leaf waxes) stimuli [5]. Frank first described the term appressorium as “spore-like organs of the fungal pathogens of plants” in 1883 [6]. Frank thought that appressoria were adhesive disc tools for a fungus to stick firmly on the leaf surface, but it was found later that many pathogens use appressoria to break through the tough outer layer of plants [7]. Most Ascomycota and Basidiomycota are able to differentiate this specialized infection–appressorium to infect their hosts. Appressoria are essential for blast diseases, powdery mildews, rusts, and devastating oomycete diseases, such as potato late blight [7].

The interaction between the host and fungi can be investigated from two sides: the process of pathogenic fungi infecting plants, and the plant’s immune response after being infected by pathogenic fungi. In excellent reviews, the response of plants in plant–pathogen interactions has been discussed in detail [8,9,10,11]. The majority of antimicrobial treatments in plants are facilitated with the purpose of prevention but not cure. Once the plant-pathogenic fungi recognize and penetrate the vegetal tissue of the host plants, it is hard to successfully eradicate infection due to the low accessibility of the therapeutic agents within the plant [12]. Fungal host attachment is a vital step of fungal pathogenesis. The management of fungal attachment is expected to provide broad-spectrum crop disease intervention [13]. Investigation into the molecular mechanisms of the appressoria formation of plant pathogens contributes to improving the understanding of the molecular mechanisms of host–fungi interactions. This review summarized the recent 5 years’ progress, which mainly determined the functions of genes in appressorium formation in some fungal species including *Magnaporthe oryzae*, *Colletotrichum* species, *Ciboria shiraiana*, *Setosphaeria turcica*, *Bipolaris maydis*, *Curvularia lunata*, *Ustilago maydis*, *Cochliobolus heterostrophus*, *Botrytis cinerea*, *Fusarium graminearum*, and *Sclerotinia sclerotiorum*. Understanding the molecular mechanisms of appressoria formation in different species can be useful in finding commonalities among different species in order to design control strategies targeting appressoria formation. The latest research progress can supplement our understanding of appressoria formation and provide ideas and point the way for the following research.

## 2. Appressoria Formation

**Figure 1:**
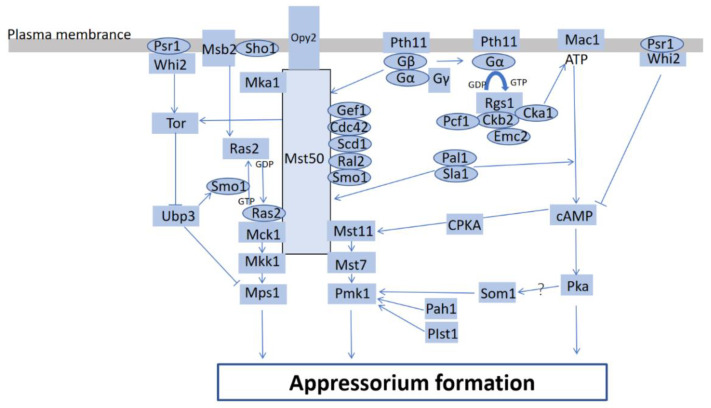
Several signaling pathways related to appressorium formation in *Magnaporthe oryzae*. The sensor proteins Msb2 and Sho1 activate the Pmk1 MAPK pathway; Whi2 interacts with Psr1, participating in the regulation of the cAMP levels and the Tor signaling pathway; Som1 functions downstream of the cAMP-PKA pathway, which may regulate the Pmk1 MAPK pathway through Som1. Rgs1 regulates G-protein signaling; the normal process of G-protein MoMagA-cAMP signaling depends on a condition of steadiness among Rgs1, Ckb2, and Emc2; Opy2 participates in the Mps1 MAPK pathway by interacting with Mst50; Ral2 interacts with Smo1, Scd1, and Mst50 to regulate the activation of the Mst11-Mst7-Pmk1 MAPK pathway by Ras2; Pmk1 pathway activation involves Mgb1, Ras2, and Cdc42; Mst50 activates Pmk1 through its interaction with Smo1, Cdc42, Scd1, Ral2; Pmk1 also requires the Mst7 and Mst11, which both bind to Mst50; Mka1, which interacts with Mst50, functions upstream of the MAPK pathway; Pal1 interacts with Sla1 and functions upstream of both cAMP and Pmk1–MAPK signaling pathways; Whi2 interacts with Psr1, participating the regulation of appressoria formation through the regulation of the cAMP levels and the target of rapamycin (Tor) signaling pathway; Ubp3 regulates GTPase-activating protein Smo1.

Spores that firmly attach to hosts germinate on the plant surface, subsequently build germination hyphae, and finally differentiate into appressoria. Appressoria will only form on surfaces with the correct physical and chemical signals [5,7]. A few natural components (cutin-derived fatty acids, wax, and volatiles emanating from plants) of the plant surface are considered chemical inducers for appressorium formation [14]. A number of important fungal signaling pathways such as heterotrimeric guanine nucleotide-binding proteins (G proteins) [15,16], cyclic adenosine monophosphate (cAMP) [17,18,19], mitogen-activated protein kinase (MAP kinases) [20,21,22,23] and calcium/calmodulin-mediated [24] signaling pathways have been extensively investigated since they are required for the formation and functioning of appressoria (Figure 1). Studies on different phytopathogenic fungi have shown that the cell cycle influences appressorium formation [12,25,26,27,28]. It seems that appressorium formation also requires cell cycle-dependent morphogenetic transitions, thereby enabling host cell penetration [25].

In addition, the transition from polarized growth to isotropic growth is necessary for appressorium formation, but penetration requires a transformation from isotropic growth to polarized growth. Polarized growth depends on the coordinated organization of cytoskeletal elements, such as microtubules and actin [7,18]. Protein features and functions are modulated not only by a sequence of the amino acids but also by post-translational modifications (PTMs). Redox modification, a post-translational modification, plays a vital role in fungal appressorium formation and invasive growth [29]. Recent studies have contributed to a better understanding of appressorium formation (Figure 1).

The interaction between plants and pathogenic fungi is very complex. These signal pathways mentioned above regulating the differentiation and development of appressorium interact with each other; however, many details of these regulatory processes and their interactions remain unknown, such as the precise surface signals sensing mechanisms. An overall understanding of the molecular mechanism of appressorium formation provides an important theoretical basis for new strategies for fungal plant disease control. Increasingly, more genes have been found to be involved in the appressorium formation of fungi, whether these new genes are associated with the co-evolution of host-microbe interactions needs further research. The following is up-to-date research on genes related to appressorium formation.

### 2.1. Magnaporthe oryzae

*Magnaporthe oryzae*, at the top of the list of “the Top 10 fungal pathogens in molecular plant pathology”, causes cereal blast disease. In the study of host–pathogen interactions, *M. oryzae* has become a pathogen model with economic significance and tractability [30]. In a recent review, the appressoria of *M. oryzae* has been discussed in detail [31]. Here, we focus on the research on the molecular mechanisms of appressoria formation over five years. MoYPEL1 is of vital importance in appressorium development in *M. oryzae*, as it participates in sensing chemical and physical signal cues for appressorium development [7]. Mopas1 is indispensable in surface sensing, the *Mopas1* deletion conidia failed to undergo appressorium formation [8]. In addition, MoRBP9 is necessary for the development of appressorium in response to hydrophobicity [9]. Interestingly, NDK1 was found to be indispensable for appressoria formation on artificial hydrophobic surfaces, but not on the plant surface [10]. Membrane-bound protein MoAfo1 was found to participate in detecting signal molecules for appressoria development. The *Moafo1* deletion mutants failed to differentiate an appressorium on artificial hydrophobic surfaces, even though treated with the well-known appressorium-inducing chemicals.

Appressorium formation is rigorously regulated by both the cAMP-dependent protein kinase A (cAMP-PKA) and Pmk1 MAPK pathways. MoSom1, a transcriptional regulator of *M. oryzae*, functions downstream of the cAMP-PKA pathway and may regulate the Pmk1 MAPK pathway through MoSom1 during the rice infection. Appressorium formation was limited due to the deleting of *MoSom1* [32]. MAPK cascades, highly conserved signal transduction pathways, are required for appressorium formation in *M. oryzae*. It has been demonstrated that the genetic program controlling appressorium development in *Podospora anserina* shares common components with the phytopathogenic fungi *M. oryzae* and *Botrytis cinerea*. In these three species, the major components of appressorium development include Nox1, Nox2, NoxR, NoxD, the Pls1 tetraspanin, and the MAPK pathway Fus3/Mpk2/Pmk1 [33,34,35]. Adaptor protein Mst50 plays an extremely important role in the MAPK cascades. Overproduction-induced pheromone-resistant protein 2 (Opy2) is a vital protein for sensing external signals in *S. cerevisiae.* MoOpy2, the homolog of Opy2 in *M. oryzae*, participates in the Mps1 MAPK pathway by interacting with the adaptor protein Mst50, and is required for appressorium penetration and pathogenesis (Figure 1). According to the biochemical characterization of ∆*MoOpy2* strains, the knockout of *MoOpy2* reduces pathogenicity due to pleiotropic defects in appressorium formation, hyphal growth, appressorium turgor generation and invasive growth and so on [36]. A recent study showed that mitogen-activated protein (MAP) kinase (MAPK) activator MoMka1, which interacts with Mst50, functioned upstream of the MAPK pathway. Target deletion of *MoMKA1* led to a significant decrease in appressoria formation [37]. A Ras-like protein PoRal2, interacted with Mst50, Scd1, and Smo1 in *Pyricularia oryzae* (synonymous with *M. oryzae*), plays a vital role in appressorium formation and infection via the Mps1 MAPK and Osm1 MAPK pathways. It has been revealed that the ∆*Poral2* mutants formed appressoria with long germ tubes during appressorium formation [38]. Septins, absent in higher plants, are a broadly conserved family of guanosine triphosphatase-binding (GTP-binding) cytoskeletal proteins [39]. In the process of appressorium development in *M. oryzae*, the septin structures are remodeled from an incipient disc into a toroidal ring [40]. The data support the idea that the transitions depend on the dynamic remodeling of the F-actin cytoskeleton in part [41]. The *Smo1* (encoding a GTPase-activating protein) deletion mutants failed to undergo septin-mediated F-actin remodeling and displayed a severe defect in the development of appressoria [42]. In addition, the deletion of *Momyo1* led to a failure in appressorium formation, as well as a significant decrease in the expression of several appressorium formation involved genes such as *Mohox7*, *Mopmk1*, *Mopth1*, *Momac1*, *Momst11*, and *Momst50* [43]. Pal1, interacted with the endocytosis protein Sla1, plays a vital role in appressorium formation and maturation. This putative endocytosis-related gene *pal1* was highly expressed in the appressorium of *M. oryzae* and regulated cAMP and the Pmk1 signaling pathway (Figure 1), thereby enabling appressorium formation and maturation to facilitate infection [17]. MoWhi2, a homolog of *Saccharomyces cerevisiae* Whi2 (Whisky2), controls appressorium formation as a vital regulator in *M. oryzae*. Further studies have demonstrated that MoWhi2 interacted with MoPsr1, participating in the regulation of appressoria formation through the regulation of the cAMP levels and the target of the rapamycin (Tor) signaling pathway (Figure 1) [44]. Small GTPase RHO2 was found to be involved in cAMP-mediated appressorium development in *M. oryzae*, although the role of MoRHO2 in the cAMP signaling pathway remains unknown [1]. *RAM1*, a farnesyltransferase β-subunit gene, was essential for appressorium formation via acting upstream of the cAMP signaling pathway [2]. It was first reported that the endoplasmic reticulum (ER) membrane complex (EMC) subunit MoEmc2 was directly linked to G-protein signaling by modulating MoCk2-mediated MoRgs1 phosphorylation. The normal process of the G-protein MoMagA-cAMP signaling depends on a condition of steadiness among MoRgs1, MoCk2, and MoEmc2, which is also essential for the appressorium formation of *M. oryzae* (Figure 1) [16]. In addition, transcription factor Pcf1 was found to directly interact with the CKb2 subunit. Pcf1 enacts its functions in the mature appressoria of *M. oryzae*, even though it is not required for the initial stage of appressorium formation. Pcf1 is degraded after phosphorylation by CK2 at the protein level via ubiquitin proteasome system (UPS) in the initial appressoria but elevated at both the transcription and protein levels in the mature appressoria [45]. The Ubp (ubiquitin-specific protease) family of deubiquitinating enzymes is essential for the pathogenicity of plant-pathogenic fungi. A study described the deubiquitinating enzyme Ubp3 regulatory mechanism during the infection process of *M. oryzae* [46]. The deletion of *Ubp3* not only emerged as severely defective appressorium turgor accumulation, resulting in the impairment of appressorial penetration but also showed defects in the glycogen and lipid metabolism during appressorium formation. These results support the idea that Ubp3 is involved in appressorium-mediated infection by regulating the de-ubiquitination of GTPase-activating protein Smo1 in *M. oryzae* [46]. About a quarter of conidia of *MoUBP4* deletion mutant failed to develop an appressorium [47]. Another deubiquitinating enzyme MoUbp8 was also found to play a role in appressoria development. The deletion of *MoUbp8* resulted in a delayed appressoria formation [48].

In addition, some genes are associated with appressorium formation by regulating other pathways. It is necessary to systematically investigate the genes involved in phospholipid biosynthesis to understand the mechanism of fungal virulence and provide potential targets for novel fungicides. *MoPAH1*, encoding a putative highly conserved phosphatidate phosphatase, is required for appressorium formation in *M*. *oryzae*. The reduced expression of differentially expressed genes (DEGs) involved in appressorium formation (*CBP1*, *CHS7*, *PTH11*, *RGA4*, *CHS1*, *COD1*, *CRF1*, and *MSN2*) was confirmed by reverse transcription quantitative PCR (RT-qPCR) analysis in the *Mopah1* mutant [23]. Overexpression of *milR236*, of which promoter sequence binds the transcription factor MoMsn2 to suppress MoHAT1, led to delay appressorium development [49]. MoAa91, a novel signaling molecule regulated by RGS (G-protein signaling) and RGS-like proteins, is involved in regulating the normal appressorium formation of *M. oryzae* [15]. Li et al. revealed that MoAa91 is negatively regulated by the MoMsn2 transcription factor and that its disruption resulted in defects in appressorium formation [15]. Phosphatase encoding gene *MoCDC14* was involved in the formation of the septum that separates the appressorium from the germ tube [50]. What is more, the calcium/calcineurin signaling pathway is required for the development and virulence of plant-pathogenic fungi. MoRCN1, a calcineurin regulator regulating the calcineurin pathway, is vital to virulence in *M. oryzae*. The deletion of *MoRCN1* strains caused defects in appressorium formation and invasive growth and resulted in a significant reduction in virulence [24]. *DUG1*, *DUG2*, and *DUG3* genes participate in a fungus-specific alternative pathway that mediates glutathione degradation. DUG3 has been described to be required in the infection cycle and vegetative growth of *M. oryzae*. The deletion of *DUG3* led to delay appressorium formation and a decrease in the severity of host infection [51]. In another study, Liu et al. [52] reported that the proper initiation of appressorium formation in *M. oryzae* required the participation of the Opr1/JA signaling pathway. In *M. oryzae*, jasmonic acid was synthesized by fungal OPDA (12-oxophytodienoic acid) reductase, MoOpr1 [52]. In the cAMP signaling defective mutants, the appressorium formation could be induced by several jasmonates (12-OPDA, JA, and Me-JA). These results indicate that fungal jasmonate plays an important role in determining the precise induction of appressorium formation [52]. The appressoria and infection hyphae of plant-pathogenic fungi enact their full functions via the high activities of peroxisomal fatty acid catabolism. Fatty acid β-oxidation and the glyoxylate cycle were unexpectedly found to provide energy for appressorium formation [53,54]. For instance, impaired fatty acid β-oxidation and glyoxylate cycle activity resulted in the decreased melanization of appressoria and reduced the integrity of the cell wall, thereby leading to reduced glycerol synthesis and increased glycerol leakage in the *pex19* deletion nonvirulent mutant of *M. oryzae* [55]. A study reported that the activation of the glyoxylate cycle possibly requires acetic acid, one of the products from the Cbp1-catalyzed conversion of chitin into chitosan, to induce appressorium differentiation [56]. eIF3k is required for modulating stress tolerance and enhancing the lifespan of *Neurospora crassa* and *Caenorhabditis elegans*. Lin and colleagues [57] identified a homolog of eIF3k in *M*. *oryzae* and emphasized that it is necessary to systematically evaluate individual subunits of the non-essential eIF3 subcomplex during host–pathogen interactions. The deletion of *MoOeif3k* resulted in the suppression of the transportation and degradation of glycogen (glucophagy) and attenuated appressorium turgor. However, the degradation of lipid bodies (lipophagy) of ∆*MoOeif3k* strains was not adversely affected during conidial germination and appressorium formation. These results indicate that the MoOeIF3k likely participated in the appressorium integrity via the selective translational regulation of proteins associated with turgor development and glycogen mobilization in *M*. *oryzae* [57].

In enhancement, many other diverse genes have been found to be involved in the formation of appressoria. For example, amino acid synthesis genes are required for appressorium formation. A recent study revealed the importance of cytoplastic MoGln2 (*M. oryzae* glutamine synthetase) in *M. oryzae*. MoGln2 not only catalyzes the synthesis of glutamine but is also required for appressorium formation in *M. oryzae* [58]. Aspartate transaminase (AST) plays its functions by catalyzing the transamination reaction between glutamate–aspartate in *M. oryzae*. *MoAST2* knockout mutants severely reduced conidiogenesis and appressorium formation. *MoAST2*-mediated pathogenesis should involve at least the contribution of arginine biosynthesis [59]. MoCpa1, an ortholog of *S. cerevisiae* Cpa1, was identified and characterized its roles in the *M. oryzae* genome. MoCpa1-mediated arginine biosynthesis is required for infection-related morphogenesis, appressorium formation, fungal development, and conidiation in *M. oryzae* [60]. That the high expression of glutamate synthase gene *MoGLT1* in appressoria indicated that the role of MoGLT1 was indispensable for appressoria formation [61]. Cell polarity plays an important role in the appressoria formation progress. A homolog of Tea1 from *Schizosaccharomyces pombe*, PoTea1, is highly dynamic during appressorium formation and localizes at hyphal tips and appressoria in *P. oryzae*. The *Potea1* deletion strain forms a long germ tube with a small appressorium, resulting in delayed appressorium differentiation. Thereby, it is speculated that PoTea1 enacts its role in appressorium formation by mediating cell polarity in *P. oryzae* [18]. An evolutionarily conserved nucleosome assembly protein (Nap1) is required for multiple cellular processes in eukaryotes. In addition, Nap1 functions as a key factor in appressoria formation with an optimum turgor pressure. A loss of *Nap1* resulted in pleiotropic defects in growth, appressorium morphology, and appressorium turgidity in *M. oryzae* [62]. *MoGAS1, MoGAS2*, and *MoMAS3-MoMAS6* encode the six Magnaporthe appressoria-specific (MAS) proteins. MoMas5 is localized to the appressoria and the penetration peg and is constitutively expressed in appressoria. At the early infection stage, weak signals of MoMas5 were observed in the penetration peg. What is more, *MoMAS3*-*MoMAS6* were expressed in appressoria as well as at the early infection stage [63]. MoVast1, a novel VASt domain protein, is anchored to the membrane system and participates in sterol homeostasis in *M. oryzae*. The *MoVAST1* disruption mutant showed impaired appressorium development, conidial defects, and attenuated pathogenicity [64]. MoIst1, a subunit of endosomal sorting complexes required for transport (ESCRT)-III, participates in appressorium development, pathogenicity, and autophagy in *M. oryzae*. The disruption of *MoIst1* resulted in a significant decrease in the sporulation and formation of appressoria [65]. The bicarbonate (HCO_3_^−^) transporter family including the anion exchanger (AE) group is required for multiple physiological processes by regulating acid–base homeostasis. The *MoAE4* deletion mutants displayed defects in conidiation, appressorium formation, and pathogenicity [66]. Fungi harbor PTEN homologs (a dual-phosphatase tumor suppressor in humans). PTEN is often dysregulated by alternative splicing in humans. Recently, an alternative splicing case was found in the PTEN homolog of *M. oryzae* (MoPTEN). The conidiation, appressorium formation, and pathogenesis of the ∆*MoPTEN* strain caused defects. MoPTEN can completely recover the *MoPTEN* deletion mutant (∆*MoPTEN*), while the defects of conidium and appressorium formation can be restored partially by MoPTEN-1 [67]. *53BP1* encodes a signaling transducer protein involved in the G2-M cell cycle checkpoint of higher eukaryotes. Mop53BP1 in *M. oryzae* was observed to be located in nuclei during the maturation of appressorium and penetration. Virulence of *Mop53BP1* deletion mutants was deduced, even though it formed more than one appressorium per conidium [68]. Targeted deletion of *MoPer1* reduced appressorium formation, and the turgor of appressorium in the ∆*Moper1* mutant was decreased, which indicated the importance of MoPer1 in appressorium formation [69]. Deletion of carbon catabolite repressor *MoCreA* severely limited the formation of appressoria [70]. The deletion of DEAH-Box protein gene *DHX35* decreased the rate of appressoria formation [71]. Deletion of *Moscad2*, mitochondria β-oxidation enzyme, resulted in abnormal appressoria formation [72]. The mutants formed abnormal appressorium because of the absence of the endoxylanase I gene *Moxyl1A* [73]. Interestingly, appressorium development will be restrained by the presence of glucose beneath the cellophane layer in the medium [33]. The accumulation of reactive oxygen (ROS) species plays a vital role in appressorium formation and maturation in *M. oryzae*. The induction of ROS around the appressorium is utilized to penetrate during the development of *M. oryzae* [74,75].

### 2.2. Colletotrichum

*Colletotrichum* species, including *C. anthracis*, *C. scovillei*, *C. fructicola*, *C. graminicola*, *C. higginsianum*, *C. higginsianum*, *Colletotrichum gloeosporioides*, and *C. higginsianum*, are well-known phytopathogenic fungi with a broad range of hosts, such as cereals, fruit plants, legumes, vegetables, fruit trees, and forest trees [76]. Among them, the appressoria formation of *C. gloeosporioides* has been most extensively studied. CgMk1 (the MAPK) was demonstrated to be essential for appressorium formation and pathogenicity in *C. gloeosporioides* [77]. Subsequently, three upstream components of CgMk1 including CgSte50, CgSte11, and CgSte7, were found to positively regulate the phosphorylation of CgMk1. The knockout of *CgSte50*, *CgSte11*, and *CgSte7* led to pleiotropic defects in appressorium formation, invasive growth, and pathogenicity, similar to the defects observed in the *CgMk1* mutant. Compared to the wild-type mutants, the deletion of *CgSte50*, *CgSte11*, *CgSte7*, and *CgMk1* resulted in the altered accumulation of reactive oxygen species (ROS) at the initial stage of appressorium formation [22]. These data support the idea that the CgMk1 MAPK cascade is essential for various key functions in *C. gloeosporioides* [22] CgMsb2, a putative sensor cooperated with CgSho1 to activate CgMk1 in *C*. *gloeosporioides*, is a key factor in the recognition of various host surface signals; *CgMsb2* deletion leads to a significant defect in appressorium formation and penetration [78]. *CgMCK1*, encoding a MAPKKK protein, knockout mutant failed to elaborate an appressorium [79]. Multiunit-flavoenzyme NADPH oxidases (NOXs), regulating signaling pathways, are required for multiple physiological functions in living cells. The roles of CgNOXA, CgNOXB, and CgNOXR, components of the NOX complex, have been described in *C. gloeosporioides*. CgNOXB and CgNOXR are involved in the appressorium formation of *C. gloeosporioides* as key factors. CgNOXB and CgNOXR not only regulate the production and distribution of ROS in hyphal tips and appressoria but also control the specialized remodeling of F-actin in hyphal tips and appressoria [80]. Rho GTPase Cdc42 was found to play role in normal appressoria formation and participate in the regulation of ROS-related genes [81]. Ethylene (ET) can be sensed by plant-pathogenic fungi to accelerate their spore germination and subsequent infection. It was demonstrated that ET was essential for appressorium development and virulence expression. ET changed the transcription in a large set of appressorium development- and virulence expression-related genes through the G protein-coupled receptor (GPCR) and MAPK pathways in *C. gloeosporioides*. In the end, ET enhanced appressoria development and ROS accumulation at the inoculation site on leaves [82]. Therefore, depending on the in-depth understanding of the exact ET sensing and signaling mechanisms in fungi, it is promising to manage the disease during fruit ripening by selectively blocking the ET perception in fungal pathogens in the future [82]. CgEnd3, an endocytosis-related protein in *C. gloeosporioides*, has been described as a promising antifungal target. CgEnd3 regulates appressorium formation and endocytosis in a calcium signaling-independent manner. *CgEnd3* deletion mutants showed deficient appressorium formation, appressorium penetration ability, and pathogenicity [83]. CgFim1, an actin cross-linking protein fimbrin homolog, was identified and characterized by its physiological functions in *C. gloeosporioides*. The deletion of *CgFim1* prevented polar growth and appressorium development due to disrupting the actin dynamics and ring structure formation in the appressorium as well as affecting the polarity of the actin cytoskeleton in the hyphal tip [84]. Arginine, one of the several types of amino acids, is required for the biochemical and physiological functions of fungi. The *CgCPS1* gene was identified experimentally to be involved in arginine biosynthesis through encoding carbamoyl phosphate synthase. The knockout of *CgCPS1* showed defects in appressorium formation, a slow growth rate, and loss of virulence in *C. gloeosporioides*, which failed to develop lesions on apple leaves and fruits [85]. CgNVF1 was found to be essential to the melanin synthesis of *C. gloeosporioides*, the *CgNVF1* deletion mutants were unable to form appressoria [86]. The *CgRGS3* knockout strain showed a significant reduction in appressoria formation [87]. The *CgCMK1* deletion mutants failed to form an appressorium [88]. Silencing of *COM1* by RNAi strategy had severely limited the differentiation of appressoria in *C. gloeosporioides* and rendered the fungus non-pathogenic [89]. Laccase gene *Cglac3* was highly expressed during the development of appressorium [90]. Only a few conidia of *CgHOS2* deletion mutants formed appressoria and the appressoria were abnormally shaped [91]. An ATP-binding cassette protein CgABCF2 was found to be significantly upregulated in appressorium development [92], and the *CgABCF2* deletion mutant failed to form appressoria [93]. CAP20, a perilipin homolog protein, plays a vital role in functional appressoria development in *C. gloeosporioides* [94].

The formation of appressoria in other subspecies has been slightly less studied, although these studies have also led to a better understanding of the mechanisms of appressoria formation. *CDA3* encoding a conserved structural domain of a polysaccharide deacetylase is involved in the formation of attachment cells of *Colletotrichum* CDA3 containing a conserved structural domain of a polysaccharide deacetylase is involved in appressoria formation of *Colletotrichum anthracis*, which is one of the most important plant-pathogenic fungi [19]. *CfPMK1* gene (Fus/Kss1 MAP kinase) knockout mutants are unable to develop an appressorium [20]. Target deletion of mitogen-activated protein kinase gene *CfMKK1* in *C. fructicola* resulted in its failure to form an appressorium [21]. The deletion of vacuolar protein sorting 39 *CfVPS39* significantly limited the development of appressoria in *C. fructicola* [22]. The knockout of the autophagy-related gene *CfATG9* decreased the rate of appressoria formation [23]. An ortholog of the FUS3/KSS1-related MAPK gene, *CfMK1* was identified and characterized by its function in *C. fructicola*. The *Cfmk1* knockout mutants caused defects in appressoria formation, leading to failure to penetrate plant epidermal cells [95]. Target deletion of *CsPMK1*, a mitogen-activated protein, totally blocked the formation of appressoria in *C. scovillei*. Moreover, CsPMK1 was found to interact with the homeobox transcription factor CsHOX7, which is required for appressorium formation [20]. RAC1, a Rho GT-Pase family member, is a highly conserved small GTP-binding protein. RAC1 functions in the fungal development and pathogenicity of several plant-pathogenic fungi. *Csrac1* deletion mutants caused abnormally shaped conidia, as well as reduced conidial germination and appressorium formation. Compared to wild-type strains, the conidia of the *Csrac1* deletion mutants were larger in size. Interestingly, the *ΔCsrac1* mutants formed the larger appressoria, but it failed to penetrate. These results provide the insight that CsRAC1 is not only required for germination and appressorium formation but also plays a vital role in the penetration and post-infection of *C. scovillei* [96]. As an adaptor protein, Csste50 was found to activate the MAPK cascades, which is required for appressoria formation. Deletion of *Csste50* completely blocked the development of appressoria in *C. scovillei* [97]. CsPOM1 enacts its key roles in appressorium formation via functioning downstream of cAMP or in a cAMP-independent manner. Only 8% of *CsPOM1* knockout mutant conidia developed appressoria [98]. Target deletion of mitochondrial protein tyrosine phosphatase gene *CgPTPM1* significantly reduced the development of appressoria in *C. graminicola* [37]. Acetyl-CoA synthetase ChAcs2 was observed highly expressed during appressorium formation. The deletion of acetyl-CoA synthetase ChAcs2 led to a delay in appressoria development [24]. The deletion of Ser/Thr kinase gene *ChSch9* in *C. higginsianum* reduced the formation of appressoria [25]. ChODC, a putative ortholog of yeast *SPE1* in *C. higginsianum*, is involved in the polyamine biosynthesis pathway. ChODC regulates appressorium function as a mediator of the cAMP and mTOR signaling pathways [19]. *CsAtf1* is a gene that encodes a bZIP transcription factor in *C. siamense*. The deletion of *CsAtf1* led to slightly retarded mycelial growth and abnormal appressorium formation [99]. The *CoHox3* knockout mutants formed abnormal appressoria and did not express the appressorium-specific gene *CoGAS*1 in *C. orbiculare* [100]. Zn(II)_2_Cys_6_ transcription factor CoMTF4 functions downstream of MOR (morphogenesis-related NDR (nuclear Dbf2-related) kinase pathway). CoMTF4 was found to be indispensable for plant-derived signals, which deduce the development of appressorium in *C. orbiculare* [101].

### 2.3. Other Fungi

The formation of appressoria in the *Magnaporthe* and *Colletotrichum* species has been studied extensively. However, it is also necessary to study the formation of adherent cells in other plant-pathogenic fungi. Within the last five years, researchers have also investigated the formation of appressoria in plant-pathogenic fungi such as *Curvularia lunata*, *Setosphaeria turcica*, *Ciboria shiraiana*, *Ustilago maydis*, *Cochliobolus heterostrophus*, *Botrytis cinerea*, *Fusarium graminearum*, and *Sclerotinia sclerotiorum.* In conclusion, all of these studies contribute to a more comprehensive understanding of appressoria formation in phytopathogenic fungi.

*Curvularia lunata* is the pathogen that causes maize curvularia leaf spot disease. Iron assimilation gene *ClFTR1* had been found to regulate the formation of appressoria [102]. Another study showed that ClM1 regulated the ClBRN1 transcriptionally and negatively regulated ClNOX2 transcriptionally, while ClNOX2 regulated the transcription of ClSCD1. Loss of *ClM1* and *ClNOX2* had a negative effect on appressoria development in *Curvularia lunata* [103]. *ClFTR1*, the iron permease gene *FTR1* in *Curvularia lunata*, positively regulated conidial germination and appressoria formation in the biotrophy-specific phase. Compared to wild-type (WT) *CX*-3, the *Clftr1* deletion mutants caused delayed conidial germination and were reduced in appressoria formation. In addition, *ΔClftr1* and *ΔClnps6* mutants showed downregulation of several genes that were responsible for conidial germination, appressoria formation, non-host selective toxin biosynthesis, and cell wall-degrading enzymes [102]. ClNOX2-derived ROS signaling pathway and the ClM1-mediated CWI signaling pathway are involved in DHN melanin biosynthesis as a cross-linked manner. Loss of *ClM1*, the cell wall integrity (CWI) mitogen-activated protein kinase gene in *Curvularia lunata*, led to virulence reduction. Northern corn leaf blight caused by *Setosphaeria turcica* is one of the important corn leaf diseases. Two members of septin proteins StSep1 and StSep4 were observed highly expressed during the formation of appressoria in *Setosphaeria turcica* [104]. Splicing factor SC35 gene *StSC1* in *Setosphaeria turcica* was highly expressed during the appressoria formation [105]. The deletion of *BmSte50*, interacting with a MAPK kinase kinase BmSte11, led to failure in appressoria formation in *Bipolaris maydis*, which causes southern leaf blight (SLB) of corn [106]. Biz1, phosphorylated by the kinase responsible for cell cycle progression, was described as a regulator for activation of the appressoria formation in the corn smut fungus *Ustilago maydis* [12]. Copper ions are required for appressorium formation. *ChCTR1* and *ChCTR4* (two copper transporter genes) play critical roles in the appressorium formation and mutation of *ChCTR1*, and *ChCTR4* suppresses the appressorium formation of *Cochliobolus heterostrophus*, the main pathogen that results in SLB of corn [107]. *Ciboria shiraiana* is the main pathogen that results in mulberry sclerotia diseases. CsXbp1 has been described as a crucial factor in appressoria formation and pathogenicity in *Ciboria shiraiana*. Compared to wild-type strains, *CsXbp1* RNA interference (RNAi) strains showed significantly delayed appressoria formation. In addition, the defects in the compound appressoria of *CsXbp1* RNAi strains led to weakened pathogenicity [108]. *CsGPA1*, a G protein α subunit gene, was identified and characterized by its roles in *Ciboria shiraiana*. The *CsGPA1*-silenced strains were significantly reduced in appressoria formation compared to the wild-type and empty vector strains [109].

In contrast to the unicellular appressoria formed by *Magnaporthe* and *Colletotrichum* species, several plant pathogens such as *Botrytis cinerea*, *Fusarium graminearum*, and *Sclerotinia sclerotiorum* develop multicellular appressoria, called infection cushions (IC) [46]. A great number of putative effector proteins (PE) are specifically upregulated in the IC of *Fusarium graminearum*. Among them, FgPE1 localized at the fungal cell wall was secreted and localized at plant cell walls close to IC during the formation of IC [46]. In addition, genes encoding carbohydrate-active enzymes (CAZymes) and secondary metabolism (SM) gene clusters are specifically upregulated in IC in *Fusarium graminearum* [46]. Another study also revealed that categories of genes such as CAZymes, putative effectors, and SM were significantly upregulated in the IC of *Botrytis cinerea* [47]. TALEN-induced knockout of *HvMPK3* significantly limited the formation of IC [48]. *Sclerotinia sclerotiorum*, a well-known necrotrophic phytopathogenic fungus, infects a wide variety of economically important crops, including oilseed rape, sunflowers, soybeans, peanuts, and lentils [110]. Saprotrophic hyphae of *Sclerotinia sclerotiorum* differentiate the multicellular appressorium (compound appressoria) during infection, thereby enabling the penetration of the cuticle and cell walls of healthy plant issues [111]. It is necessary to systematically investigate the appressoria formation and development of *Sclerotinia sclerotiorum* because the synthesis of cell wall-degrading enzymes, effector proteins, and secondary metabolites in appressorium make it a powerful arsenal [112,113]. SsFoxE3 is one of the forkhead box family transcription factors (FOX TFs) members in *Sclerotinia sclerotiorum*. It was reported that *SsFoxE3* disruption resulted in the reduced formation and developmental retardation of compound appressoria, which caused the loss of pathogenicity [114]. Cox17 plays an important role in shuttling cooper from the cytosol to the mitochondria for the cytochrome coxidase (CCO) assembly. A recent study found that *SsCox17* gene-silenced strains showed impaired activity in appressoria formation, indicating that SsCox17 was important for appressoria formation in *Sclerotinia sclerotiorum* [115]. In *M. oryzae*, the *Magnaporthe* appressoria-specific (MAS) proteins are required as key factors in appressorium formation. *Sscnd1*, a MAS homolog gene, is highly induced at the early infection stage of *Sclerotinia sclerotiorum*. At the penetration phase of *Sclerotinia sclerotiorum*, Sscnd1 is also essential for compound appressorium formation and fungal full virulence. The upregulated expression of *Sscnd1* was detected at the early infection stage. The gene-silenced strains of *Sscnd1* displayed a significant reduction in appressorium formation [116]. The SsATG8 in *Sclerotinia sclerotiorum*, one of the core components of the autophagy machinery, and its interactor SsNBR1 (an autophagy cargo receptor) were described as a crucial factor in compound appressoria development and virulence through functional genomic approaches [117]. Yu et al. investigated the physiological functions of two components of cAMP signaling, including SsPKA and SsPKAR. The SsPKAR played a vital role in carbohydrate metabolism and mobilization, which are essential for the appressorium development of *Sclerotinia sclerotiorum*. Defects in sugar alcohol metabolism defects resulted in an inability to form appressoria and sclerotia [118]. FKH, a forkhead box (FOX)-containing protein, enacts its function by regulating transcription and signal transduction. A study described SsFkh1 as a key factor in the maintenance of cell wall integrity (CWI) and the MAPK signaling pathway. SsFkh1 likely enacts its functions downstream of the substrate of SsMkk1 and is involved in appressorium development and pathogenicity in *Sclerotinia sclerotiorum* [119]. Using RNAi technology downregulation of *Ssams2*, a cell-cycle-regulated GATA transcription factor that plays a critical role in chromosome segregation, resulted in a defect in appressoria formation of *Sclerotinia sclerotiorum* [49]. In addition, the GATA-type transcription factor SsNsd1 was also involved in the appressorium development [50], and the *Nsd1* deletion mutants formed appressorium without pigment [51]. The *SsAtg1* (autophagy-related gene) disruption mutants showed defective appressoria formation [52].

The formation of appressoria is an extremely complex process, and the research on the formation of appressoria in different species can improve our understanding of the molecular mechanism of appressoria formation. Both *Magnaporthe* and *Colletotrichum* species, which form unicellular appressoria, and species such as *Sclerotinia sclerotiorum*, which form compound appressoria IC, share some common pathways in the formation of adherent cells, such as the MAPK and cAMP signaling pathways. In addition to the *NOX* genes associated with the ROS signaling pathway observed in the well-studied *Magnaporthe* and *Colletotrichum* species, similar results were obtained in the less-studied *Curvularia lunata*. Septin proteins, which are essential for appressoria development in *M. oryzae*, also apply to *Setosphaeria turcica*. Ste50 and Ste11 in *Bipolaris maydis* are also indispensable for developing an appressorium. Although there are differences in the formation of appressoria between species, there must be some consistency because they all differentiate appressoria to infest hosts. Knowledge of appressoria formation in different pathogenic fungi helps us to discover shared pathways and design control strategies that target appressoria formation.

## 3. Turgor Generation and Penetration

The morphology of appressoria is highly variable: it varies from a single-celled structure to multicellular compound appressoria, which collectively form infection cushions [120]. Thus, appressoria can be classified into two major groups: single-celled and compound appressoria [6]. Single-celled appressoria are the most common type among many species and occur at the end of germ tubes. These single-celled appressoria are of different shapes, including hook-shaped in *Blumeria Graminis* [121]; dome-shaped in *Oxydothis* species [122]; lobed in *Erysiphe* and *Neoerysiphe* species [123] and *Colletotrichum boninense* [124]; elongated and nipple-shaped in *Phyllactinia* species [125]; sickle-shaped in *Phythium* species [126], dome-shaped in *M. oryzae* [127] and irregular-shaped in *Oxydothis garethjonesii* [122].

Once formed, appressoria adhere firmly to the host surface and subsequently secrete extracellular enzymes or produce physical force (or use a combination of both factors) to facilitate cuticle penetration. A mechanical pressure toward the surface and a penetration peg is used by appressoria, thereby enabling cuticle penetration [128,129]. However, pressurized appressoria formed by the *Clu5a* gene deletion mutants were impaired in the penetration pores formation of *Colletotrichum graminicola* [130]. In order to penetrate the plant cuticle, the appressorium generates enormous pressure of up to 8.0 MPa (80 atmospheres) in *M. oryzae* [131]. This raises the question of how appressoria generate such great pressure. The appressorium has a differentiated cell wall that is rich in chitin and contains a distinct layer of melanin between the cell wall and the cell membrane [132]. Melanin is derived from a polyketide precursor [133]. Melanin biosynthesis is required for efficient glycerol accumulation [134]. The melanin layer forms an effective semipermeable barrier, which water can diffuse across, but blocks ions and other small molecules from moving into or out of the cell. Lipids, polysaccharides, and proteins were demonstrated to be the components of appressorial surface extracts in *M. grisea*. In addition, glycerol was detected by gas-liquid chromatography as the most abundant solute in appressoria, reaching a concentration of approximately 3 M [134]. Glycerol is generated rapidly during germination and germ tube elongation and decreases at the onset of appressorium formation but rises sharply during turgor generation [134]. In the appressoria of *M. grisea*, glycogen and lipid were demonstrated as the principal precursors for glycerol synthesis [135]. Significant amounts of water, which can travel into the appressorium through the melanin layer, are required for keeping such a high concentration of polyols in the solution. When water is available [5], enormous turgor pressure is generated, up to 8.0 MPa [131], and the glycerol cannot escape the melanin layer. It has been demonstrated that Sln1, a sensor kinase, controls the turgor-driven infection in *M. oryzae*. Once the turgor reaches a threshold, Sln1 negatively regulates melanin biosynthesis and the cAMP/PKA pathway [136]. Another research showed that the deletion of *Mosec61β* in *M. oryzae* decreased the turgor pressure of appressoria [137]. It has long been believed that melanin in the appressorium maintains turgor pressure by lowering the porosity of the appressorium cell wall. Interestingly, it has been reported that in *C. graminicola*, the penetration of intact leaves and artificial substrates still occurs even when melanin biosynthesis is inhibited [138]. The *Cgscd1* deletion mutants formed albino appressoria, which failed to infect plants [139]. In addition, the deletion of polyketide synthase gene *CgPks1* in *C. gloeosporioides* abolished the deposition of melanin in appressoria, which resulted in insufficient turgor pressure and pathogenicity reduction [140]. A 1,3,8-trihydroxy- naphthalene reductase, Buf1, has been found to be essential for melanin synthesis as a rate-limiting enzyme in *Pyricularia oryzae* 70–15 [141]. Melanin is critical for appressorium function in many species [142], but there are exceptions. For example, evidence has revealed that a high turgor of up to 5.13 MPa, could also be observed in the non-melanized appressoria of *Phakopsora pachyrhizi* [143]. Thus, melanin possibly does not play the same function in other non-melanized fungi, which may still take on mechanical appressorium-mediated infection [144]. It has been demonstrated that the non-melanin-pigmented appressorium from *Erysiphe graminis* f. sp. *hordei* also generates turgor pressure of approximately 2–4 MPa. In addition, during appressorium penetration by *E. graminis*, the presence of extracellular enzymes, such as cellobiohydrolase and cutinase, was observed [145].

**Figure 2:**
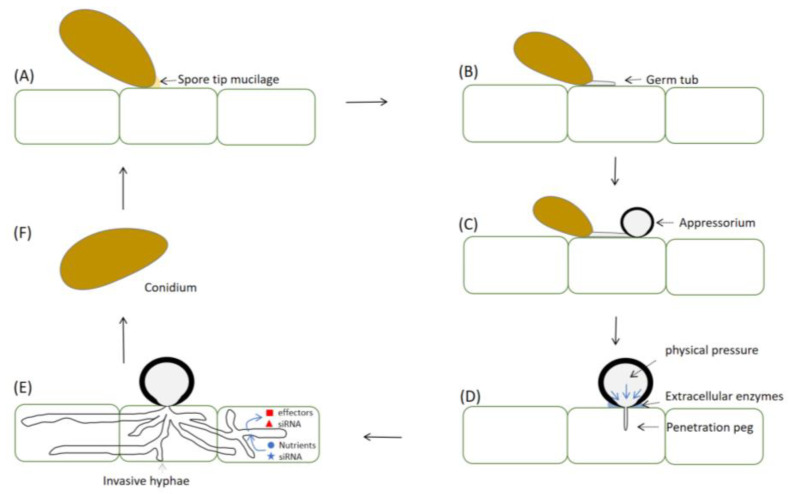
Concept of plant infection by appressorium-forming fungi. (**A**): A spore secretes an adhesive extracellular matrix that allows it to attach firmly to the host surface, then the infection begins. (**B**,**C**): Subsequently, the spore tapering end develops a single, polarized hypha, which grows along the host plant surface, and finally differentiates into an appressorium. (**D**): Formed appressoria adhere firmly to the host surface and subsequently secrete extracellular enzymes or produce physical force (or use a combination of both factors) to facilitate cuticle penetration. Cellular turgor is translated into mechanical force by penetration peg, forcing it through the leaf cuticle. (**E**): The pegs differentiate invasive hyphae to colonize rapidly epidermal and mesophyll tissues and secrete effectors and sequester nutrients from living host cells. The fungus ramifies intra- and intercellularly in susceptible host tissue upon penetration. Lesion formation is initiated with host cell death starting 4 to 5 days after infection. (**F**): The next generation of conidia is then produced.

In summary, these results demonstrate that a combination of enzymatic activity and mechanical force influences host penetration by *E. graminis*. At the base of the appressorium, in contact with the plant cuticle, is the appressorium pore, with a diameter of approximately 5–10 microns, where the cell wall is extremely thin. Meanwhile, the penetration peg diameter is in the range of 0.5 microns, focusing the turgor force generated in the appressorium on an extremely tiny area of the host cell surface [133]. Cellular turgor is translated into mechanical force, which is exerted by the emerging penetration peg, forcing it through the leaf cuticle. Subsequently, the invasive hyphae, differentiated from pegs, rapidly colonize epidermal and mesophyll tissues, secrete effector proteins and sequester nutrients from living host cells. The fungus ramifies intra- and intercellularly in susceptible host tissue upon penetration [146]. Lesion formation is initiated with host cell death starting 4 to 5 days after infection. The next generation of conidia is then produced [5] (Figure 2).

Both melanized and non-melanized appressoria can generate huge turgor to facilitate penetration, which is completed by applying pressure to the plant cuticle. Therefore, there should be similarities and differences in pressure generation in different types of appressoria. Melanin is indispensable in the turgor generation in melanized appressoria. However, how does a non-melanized appressorium complete the pressure generation? Understanding the common mechanism of turgor generation shared by different appressoria improves the understanding of the appressorium formation mechanism, which significantly promotes the prevention and management of appressoria-meditated plant fungal diseases.

## 4. Factors Limiting Appressorium Formation and Their Potential Applications against Fungal Diseases of Plants

Researchers are all making unswerving efforts to discover agents with high efficiency and safety. Chemical Synthesis is one of the strategies for developing fungicides. Mefentrifluconazole, a novel triazole fungicide, reduces appressorium formation and sporulation, results in the abnormal development of appressoria, and eventually affects *C. scovillei* infection on pepper [147]. A few biomacromolecules, metabolites, and microorganisms were found to limit the formation of appressorium of fungi, with potential applications in plant protection.

Biomacromolecules with biological activity have been found to limit the formation of appressorium. The vital role of β-1,3-glucanase, one of the pathogenesis-related protein (PR)-2 family members, has been described in plant defense. Evidence has shown that β-1,3-glucanase Gns6 from rice effectively inhibited the formation of germ tubes and appressoria in *M. oryzae*. In addition, the inhibition was dose-dependent, with a minimum effective Gns6 concentration of 0.3 μg/μL. As the concentration of Gns6 increased to 0.5 μg/μL, the rate of germ tube formation decreased from 100% to 16%, while appressorium formation was fully inhibited [148]. A GroEL protein, separated and purified from photosynthetic bacteria *Rhodopseudomonas palustris*, had an obvious antagonistic effect on the appressorial formation and pathogenicity of *M. oryzae*. This evidence shows that this photosynthetic bacterium could be a potential biocontrol agent against rice blast control [149]. Cellulose, an organic polysaccharide, has been investigated and applied in different fields [150]. *Phakopsora pachyrhizi* is the causative agent of Asian soybean rust, which is the most devastating soybean production disease worldwide. The germ tubes and appressoria formation can be hindered with cellulose nanofibers (CNFs) covering soybean leaves [151].

Natural products are one of the resources for discovering novel fungicides. Metabolites limiting the formation of appressorium have contributed to fungicide designing and development. Pachybasin, isolated from *Ascochyta lentis*, can potently inhibit appressoria formation of both *Uromyces pisi* on pea and *Puccinia coronata* f. sp. *avenae* on oat, which indicates that pachybasin could be a very promising molecule with effective potential as an antifungal agent against both rust and powdery mildew in pea and oat, respectively [152]. Tangeretin, a natural flavonoid, is mainly extracted from citrus peel. It has been reported that tangeretin effectively interferes with the appressorium formation of *M. oryzae* and completely blocks rice blast disease in seedlings under laboratory conditions, primarily because of its antioxidant activity. Mechanistically, during appressorium formation, tangeretin likely lessens lipid peroxidation within conidial cells through targeting Nox-mediated lipid peroxidation or via directly affecting Nox2 localization and/or activity alternatively during appressorium formation [153]. Metabolites from microorganisms could be potential candidates, for a large number of known antibiotics are produced by microorganisms. The natural compound antimycin A, isolated from a marine *Streptomyces* sp., exhibited inhibitory effects against the formation of appressoria, implying that the *Streptomyces* sp. can be a promising biological agent against wheat blast caused by *M. oryzae Triticum (MoT*) pathotype [154]. ZW10 strain, isolated and identified as *Bacillus velezensis*, showed a significant inhibitory effect against *M. oryzae*. Conidia showed a prolonged germination time upon being treated with the antifungal substances, including extract of fermentation broth of ZW10; 78% of the conidia could not form appressorium [155]. The sterilized culture filtrate of (1 × 10^7^ CFU mL^−1^) *Bacillus subtilis* DL76, isolated from the rice rhizosphere in a field, suppressed the conidia germination and appressorium formation of *M. oryzae*. *Bacillus subtilis* DL76 significantly contributed as a candidate biological control agent against *M. oryzae* [156]. *Bacillus subtilis* KLBMPGC81 supernatant, obtained by centrifugation and filtration through a 0.22 µm biofilter when the OD_600_ values reached 1.0–1.2, impaired appressorium formation in *M. oryzae.* A supernatant of *B. subtilis* KLBMPGC81 reduced the phosphorylation of Mps1 and Pmk1 and enhanced the phosphorylation of Cdc2 Tyr 15 to reduce the activity of the cyclin-dependent kinase Cdc2. Disruption of the MAPK signaling pathway led to abnormal cytoskeleton assembly, which is necessary for the formation of a functional appressorium [157].

The side effects of chemical fungicides on human health and the environment have attracted wide attention. Using biological control agents (BCAs) is a particularly safe and sustainable technology. Kinds of microorganisms were found to have the potential to become BCAS against appressoria-meditated plant fungal diseases. Strain BS1 was isolated from rhizosphere soil in a pepper field and was identified as *B. velezensis*. The BS1 suspension at concentrations of 4 × 10^7^ CFU mL^−1^ not only showed inhibitory effects against the growth, appressorium formation, and disease development of *C. scovillei* through consistently producing cellulase, protease, and siderophore but also promoted chili pepper seedling growth, even though the growth promotion mechanism remains to be determined [158]. Culture filtrate of *Streptomyces griseorubiginosus* LJS06 impaired the membrane integrity and energy metabolism of *Colletotrichum orbiculare* and induced reactive oxygen species accumulation, which led to inhibited conidial germination and appressorium formation [159]. The severity of cucumber anthracnose caused by *C. orbiculare* was significantly reduced by the spore suspension (1 × 10^8^ CFU mL^−1^) of LJS06. The LJS06 may also colonize or enter the plant tissues by producing extracellular enzymes (cellulase, chitinase, and pectinase) to induce disease resistance in the plant host [159]. A recent study showed that *B. subtilis* at concentrations of 4 × 10^5^ CFU mL^−1^ could inhibit the normal development of appressorium of *Blumeria graminis* f. sp. *tritici* [160]. Transcriptome analysis results suggest that *B. subtilis* may induce disease resistance related to the salicylic acid-dependent signal pathway in wheat [160].

Those potential factors limiting appressorium formation are under experimental conditions. Therefore, further experiments are needed to evaluate the effects of these candidates in a complex natural environment. In addition, the molecular mechanism of the candidate inhibiting the appressorium formation requires more concern. A full understanding of such mechanisms can further the development and application of plant fungal disease fungicides that target appressorium formation.

## 5. Summary

Fungi are a highly diverse group of plant pathogens with a huge impact on agriculture. Fungi have a wide range of lifestyles and high genetic flexibility, thereby enabling rapid invasion to new hosts, the development of resistance to fungicide treatments, and disruption to the R gene-mediated resistance of plants [1]. Thus, fungal disease management will remain a huge challenge in plant pathology for a long time. A deep understanding of the molecular mechanisms of host–pathogen interactions is paramount for improving the current crop protection strategies or designing novel measures for disease control. Fungal host attachment and penetration are the key steps of fungal pathogenesis. The pathogens must first settle on the plant surface to accomplish infection and overcome the counterforce of infection pressure from the plant surface. For many fungi, the specialized infection structures appressoria are differentiated to promote this purpose. Therefore, appressoria are qualified to provide critical sites for therapeutic intervention. After the plant-pathogenic fungi have recognized and penetrated the host vegetal tissue, it is almost impossible to eradicate infection due to the low accessibility of the therapeutic agents within the plant. Therefore, interfering with initial plant infection becomes one of the best means to manage the most economically significant plant diseases. With the advancement of various technologies, we have gained a more in-depth and comprehensive understanding of the molecular mechanism of appressoria formation. An in-depth understanding of the molecular mechanisms required for the formation processes of appressoria will provide an unprecedented opportunity for plant pathology research and the improvement of agriculture practices. For example, fungicide interferes with the melanin biosynthesis of fungi, thus producing non-functional or less effective appressoria [161]. This review focuses on the up-to-date developments that provide a new understanding of the molecular mechanisms regulating the induction and function of appressoria. In addition, studies have found a number of biological control agents that work on appressoria formation. The production of antimicrobial secondary metabolites with inhibiting effects against pathogens is one of the direct modes of action by BCAs [162]. However, the mode of action of BCAs is complex [163], and whether they act via nutrient competition, inducing resistance, or other mechanisms needs further research. By combining the research on biological control agents with the molecular mechanisms of appressoria formation, plant fungal diseases are expected to be controlled at the attachment stage in the future.

Although the mechanism of appressoria formation has become clearer with extensive research, there is still a lot that remains unknown. For instance, the precise mechanisms by which surface signals are sensed are far from clear. What is the mechanism of the non-melanized appressoria-generated turgor? How does the appressorium penetrate the host surface with such enormous pressure without being damaged? What is the mechanism that ensures that a peg emerges from the side in contact with the plant cuticle? In addition, even though appressoria were first observed in plant pathogens, they have also been observed in saprobes [34], insect-pathogenic fungi [164], and symbionts. How does an appressorium function in other organisms? Furthermore, there are several similarities in the appressoria of plant-pathogenic fungi and traps of nematode-trapping fungi [165]. Can the discoveries of the appressorium of plant-pathogenic fungi be applied to the traps of nematode-trapping fungi? Much of what we know has been gained from a very small number of fungal species, and this detailed review will be helpful to further the understanding of the precise mechanisms of appressoria formation.

## Data Availability

Not applicable.
